# Sensing the Anti-Epileptic Drug Perampanel with Paper-Based Spinning SERS Substrates

**DOI:** 10.3390/molecules27010030

**Published:** 2021-12-22

**Authors:** Andrea Macrelli, Nicolò Simone Villa, Andrea Lucotti, David Dellasega, Paolo Maria Ossi, Matteo Tommasini

**Affiliations:** 1 Department of Energy, Politecnico di Milano, 20133 Milan, Italy; andrea.macrelli@polimi.it (A.M.); david.dellasega@polimi.it (D.D.); paolo.ossi@polimi.it (P.M.O.); 2 Department of Chemistry, Materials and Chemical Engineering “G. Natta”, Politecnico di Milano, 20133 Milan, Italy; nicolosimone.villa@polimi.it (N.S.V.); andrea.lucotti@polimi.it (A.L.)

**Keywords:** Perampanel, SERS, spinning cell, Lee-Meisel colloids

## Abstract

The applications of SERS in therapeutic drug monitoring, or other fields of analytical chemistry, require the availability of sensitive sensors and experimental approaches that can be implemented in affordable ways. In this contribution, we show the production of cost-effective SERS sensors obtained by depositing Lee-Meisel Ag colloids on filter paper either by natural sedimentation or centrifugation. We have characterized the morphological and plasmonic features of the sensors by optical microscopy, SEM, and UV-Vis spectroscopy. Such sensors can be used to quantify by SERS the anti-epileptic drug Perampanel (in the concentration range 1 × 10^−4^–5 × 10^−6^ M) by spinning them during the micro-Raman measurements on the top of a custom device obtained from spare part hard disk drives. This approach minimizes laser-induced heating effects and allows averaging over the spatial non-uniformity of the sensor.

## 1. Introduction

Surface-Enhanced Raman Spectroscopy (SERS) is a technique that effectively combines the spectroscopic fingerprint provided by Raman spectroscopy with a strong signal intensification when molecules are in the proximity of properly designed metal nanostructures [1,2]. In recent times, SERS has been emerging as a widely available analytical technique, and it is promisingly applied to the detection, identification, and quantification of analytes in many fields, including analytical chemistry, biochemistry, cultural heritage, forensic sciences, and trace analysis of drugs, medicines, explosives, food additives, and contaminants [1,3]. More recently, SERS was proposed as a complementary technique for therapeutic drug monitoring (TDM) of drugs characterized by a narrow therapeutic index (NTI), such as anti-cancer and anti-epileptic drugs (AEDs), for which the quantification of their concentration in blood plasma is essential to individualize the therapy and to minimize adverse effects [4,5]. Indeed, the NTI condition is defined as the restricted concentration range in which, for a given patient, a drug is effective without adverse or toxic effects [5]. Good results have been achieved so far, but the routine application of SERS in clinical practice is still unfeasible [4]. Previous works focused on the potential of SERS in TDM of selected drugs, such as Carbamazepine (CBZ) [5,6], Lamotrigine (LTG) [7], Perampanel (PER) [5,8,9,10], Apomorphine (APO) [11,12], and 6-Mercaptopurine (6-MP) [13], which are dissolved in simple solutions at concentrations within or outside their therapeutic range.

The AED selected for this work (PER) is an orally active, highly selective, non-competitive AMPA receptor antagonist authorized as an adjunctive therapy for the treatment of partial-onset seizures in epileptic patients aged 12 years and older in more than 55 countries [14,15]. Focusing on PER detection, gold and silver nanoparticles (NPs) produced by chemical methods and deposited onto glass supports, and nanostructured films produced by pulsed laser ablation (PLA) or pulsed laser ablation in liquid (PLAL) were employed as SERS substrates in the last few years [6,8,9,10,16,17]. Important issues concerning the sensitivity, the detection limit, the spatial uniformity, and the repeatability were systematically addressed.

In this work, we investigate as alternative SERS substrate, the so-called plasmonic paper [18] (i.e., a paper-based material loaded with metal NPs for plasmonic applications) that can be produced with fast, easy, and cheap methods. The first use of paper-based substrates of SERS relevance dates back to 1984 [19,20,21]. Many paper-based materials were considered, including filter paper [21,22], sand paper [23], office paper [18], newspaper, kraft paper [24], and nanocomposite cellulosic materials [25,26]. Cellulose is the most abundant biopolymer that combines biocompatibility and biodegradability with low cost, proving to be both a cheap and easy-to-use solid platform for SERS and a green reducing and stabilizing agent in the synthesis of metal NPs [21,27]. Unlike traditional rigid supports such as glass, silicon wafers, and aluminum, paper has unique features such as flexibility, porosity, thinness, ease of functionalization, wicking capability, large surface area, and 3D fibrous structure that make it an ideal platform to host NPs and to produce SERS sensors suitable for point-of-care medical diagnostics, water quality assessment and food safety [18,21,27].

The sensitivity of the paper-based SERS substrates depends on several factors, but detection limits as low as 10^−16^ M of Rhodamine 6G were reported [28]. Moreover, many methods can be employed to load the paper with metal NPs [21], such as drop casting [18,29,30], dip or immersion coating [22,31,32], in situ coating [33,34] (including silver mirror reaction [28]), inkjet printing [35,36], screen printing [37,38], spray coating [39], brushing [40], thermal evaporation [41], pen-on-paper [42], and physical vapor deposition [24], each providing the final substrate with specific plasmonic properties.

The successful use of paper-based SERS sensors for TDM has been reported for the anti-neoplastic Methotrexate (MTX) in human serum [43], for the anticonvulsant Phenobarbital (PB) in tear fluids [44], and for the antifungal Flucytosine (5FC) in spiked and undiluted serum samples [45], all in their therapeutic ranges.

In this work, we selected a rapid, simple, and inexpensive bottom–up approach to fabricate paper-based SERS sensors, i.e., dip coating of filter paper with concentrated Lee–Meisel silver colloids. To foster the production process, we used a centrifuge that accelerates the aggregation of the metal NPs on the paper fibers. In this way, starting from the same colloidal dispersion, we succeeded in producing some tens of comparable SERS substrates in just a few hours. Such substrates were applied to the qualitative and quantitative detection of PER. The measured average total and free (non-protein bound) concentrations of PER are, respectively, 343.02 and 1.53 ng/mL (about 9.8 × 10^−7^ M and 4.4 × 10^−9^ M) in plasma, and 9.74 and 2.83 ng/mL (about 2.8 × 10^−8^ M and 8.1 × 10^−9^ M) in saliva [46]. Such low concentrations were not practical for our preliminary tests on newly developed SERS substrates; therefore, we restricted ourselves to the concentration range between 1 × 10^−6^ M and 1 × 10^−4^ M.

Our work primarily aims at demonstrating the feasibility of simple production methods to fabricate a large number of SERS substrates with comparable performances, providing a quantitative response in a concentration range sufficiently close to the reference therapeutic range of PER in plasma (3 × 10^−7^ M–3 × 10^−6^ M [10]). The morphological and plasmonic properties of the sensors were carefully characterized, and important issues were assessed regarding the optimization of the detection conditions, the reduction of the background, the choice of the laser excitation, and the spatial uniformity of the sensors. In particular, the poor intra-sensor spatial uniformity was mitigated by using a spinning-cell setup (described later) that enabled us to perform quantitative measurements on paper-based sensors.

## 2. Experimental

### 2.1. Ag Colloids

We prepared silver colloids according to the procedure (c) reported in [47], which is known as the Lee–Meisel method. The metallic precursor is silver nitrate (AgNO_3_ 99.8–100%), while the reducing agent/stabilizer is trisodium citrate, TSC (C_6_H_5_Na_3_O_7_ · 2H_2_O 99.0%). Both chemicals were purchased from Sigma-Aldrich (Merck Life Science S.r.l., Via Monte Rosa 39, 20149, Milan, Italy). The procedure for the preparation of the silver colloids is summarized as follows: 72 mg of AgNO_3_ were dissolved in 400 mL of distilled water into a glass flask; 80 mg of TSC were dissolved in 8 mL of distilled water into a glass beaker to obtain 1% TSC concentration. Both solutions were separately heated up on a heating plate and mixed when the boiling point was reached. The resulting colloid was kept boiling for 45 min to promote the reaction, and it was continuously agitated through a magnetic stirrer. The solution was initially uncolored and transparent, but as soon as the NPs started nucleating and growing, it turned greenish yellow with a dull appearance. At the end of the reaction, the solution was cooled down to room temperature while keeping the stirring.

**Figure 1:**
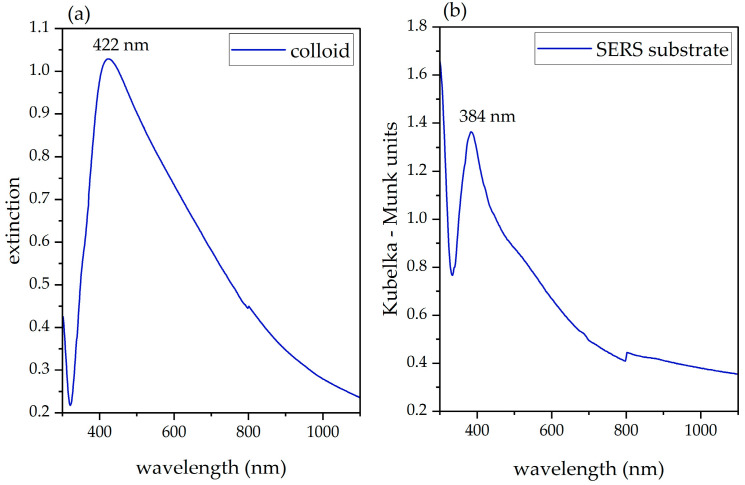
(**a**) UV-Vis extinction spectrum of the concentrated Lee-Meisel silver colloid (diluted 1:50 in distilled water to avoid optical saturation). (**b**) UV-Vis diffuse reflectance spectrum of one of the paper sensors produced by centrifugation (Section 2.2) after Kubelka-Munk conversion.

Once cooled, we concentrated the colloid by means of a centrifuge (Hettich Eba 21); six glass vials were filled with 8 mL of colloid and centrifuged for 10 min at 5000 RPM. Large and heavy NPs and aggregates settled at the bottom of the vials, while the supernatant became clear and transparent. The supernatant of each vial was gradually removed (ca. 7.5 mL), while the remaining colloid (ca. 0.5 mL) was agitated and collected for later use. We characterized the silver colloid by means of UV-Vis spectroscopy, and we recorded the spectra using a Jasco V-570 spectrophotometer. The UV-Vis extinction spectrum of the concentrated colloid peaked at 422 nm (Figure 1), but it exhibits a broad tail up to 800–1000 nm that indicates the presence of aggregates and/or nanorods [7].

### 2.2. Paper-Based SERS Substrates

We obtained a first set of SERS substrates by the natural sedimentation of Ag NPs on filter paper, slightly modifying the dip-coating procedure reported in [22]. The filter paper was purchased from Colaver s.r.l. (Via dell’Artigianato 23, 20090 Vimodrone, Milan, Italy) in the form of 50 × 50 cm^2^ sheets, with a grammage of 67 g/m^2^ and a thickness of 135 µm.

Pieces of 4 × 5 mm^2^ of paper were cut out of the sheet and thoroughly cleaned with water and ethanol to remove, respectively, undesired salts and organic contaminants. Then, they were dried at room temperature under a fume hood. The pieces of paper were placed on the bottom of 3 mL glass vials and covered with 1 mL of concentrated silver colloid previously obtained by centrifugation (see above). The vials were stocked for 3 days at room temperature to promote the natural sedimentation of the NPs on the paper. After the incubation, the supernatant and the excess of colloid were carefully removed without touching the substrates, which were dried for one hour under the hood and stored in air until their use.

**Figure 2:**
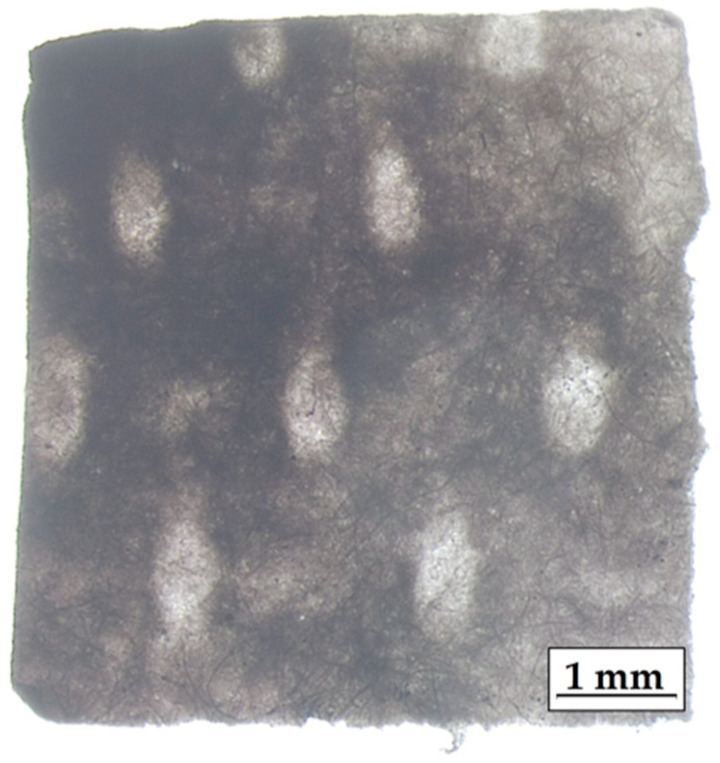
Optical image of a SERS sensor produced by natural sedimentation (0.7×).

We characterized the sensors by optical microscopy using the Olympus SZX16 Stereomicroscope in the WIBIDI Lab (Energy Dept., Politecnico di Milano), which clearly revealed the fibrous structure of paper, the dark stain of silver, and a 3D pattern of imprints (Figure 2).

We also prepared a second set of SERS substrates by a centrifuge-assisted procedure as follows. After cutting, cleaning, and drying the filter paper as described before, we placed the 4 × 5 mm^2^ pieces on the bottom of 8 mL glass vials, and we covered them with 2 mL of concentrated colloid. The vials, each containing one piece of paper, were centrifuged in swing-out rotors at 5000 RPM to exploit the centrifugal force to directly press the NPs onto the paper. We tested both 15 and 20 min of centrifugation time, but no significant differences in the SERS results were observed. Therefore, all the following spectra refer to substrates produced during a 15 min centrifugation process (unless otherwise specified). At the end of the centrifugation, after removing the transparent supernatant, the SERS substrates were extracted and dried under a fume hood.

**Figure 3:**
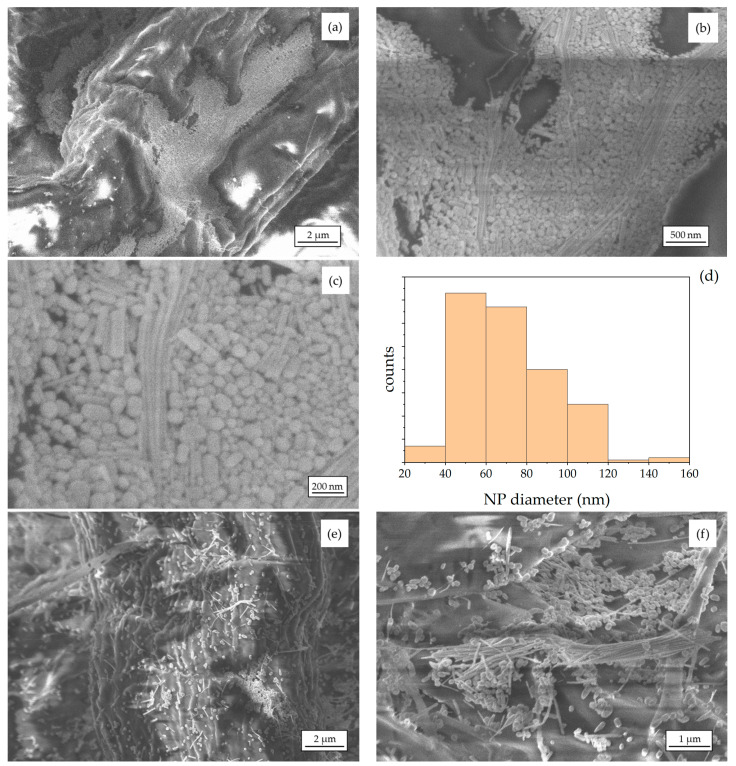
(**a**–**c**) Representative SEM micrographs of the Ag NPs and nanorods assemblies formed on the paper substrates produced by centrifugation. In (**a**), we clearly recognize one paper fiber running across the diagonal of the image. High-magnification optical images (not shown here) of the paper-based SERS sensor reveal that the average diameter of the paper fibers is a few tens of μm. (**d**) Size distribution histogram of the spherical Ag NPs sampled from image (**b**). (**e**,**f**) Representative SEM micrographs of the Ag NPs and nanorods assemblies formed on the paper substrates produced by natural sedimentation.

We employed Scanning Electron Microscopy (SEM) to investigate the aggregates of Ag NPs that form on the filter paper. All the images were acquired in the NanoLab (Energy Dept., Politecnico di Milano) by a Zeiss Supra 40 Field-Emission Scanning Electron Microscope (FE-SEM), operating in high-vacuum and equipped with the GEMINI column. Representative SEM micrographs of one substrate produced by centrifugation are shown in Figure 3a–c. Among the paper fibers, we see not only spherical NPs but also bundles of self-assembled nanorods. The particle size distribution was estimated from the SEM micrographs of Figure 3b, and its histogram is reported in Figure 3d. The average diameter of the spherical NPs is ca. 70 nm. For comparison, the SEM micrographs of one substrate produced by natural sedimentation are reported in Figure 3e,f. Remarkably, the Ag NPs and nanorods do not accumulate in the cavities of the fibers, indicating a difference in what happens during the centrifuge-assisted procedure. Instead, the Ag nanostructures are scattered around the fiber surface. We can still distinguish some aggregates of NPs and bundles of rods that are localized in specific regions of the substrate (Figure 3f). It should be mentioned that the SEM micrographs of Figure 3e,f were collected one year after the production: although the substrates were stored in a laboratory cabinet, without adopting specific measures aimed at maintaining them unaltered, the morphology is preserved, and Ag nanostructures are still visible among the fibers.

We characterized the plasmonic properties of the dried sensors produced by centrifugation by means of UV-Vis reflectance spectroscopy, using the integrating sphere setup of the Jasco V-570 spectrophotometer. The diffuse reflectance spectrum is shown in Figure 1b. The curve resembles the extinction spectrum of the concentrated colloid, blue-shifted with a peak at 384 nm, a shoulder around 500 nm, and a broad tail up to 800 nm. Both the 532 nm and the 632.8 nm laser lines may be used for an effective excitation of the plasmon.

### 2.3. Raman Spectroscopy

All the SERS spectra were collected using the dispersive Raman spectrometer Horiba Jobin Yvon LabRAM HR8000, equipped with a 600 grooves mm^−1^ grating, an Olympus BX41 microscope, and a Peltier-cooled CCD detector. Two excitation radiations were employed, namely a 532 nm laser from a frequency-doubled Nd:YAG source and a 632.8 nm laser from a He:Ne source. The spectra were analyzed with the Omnic™ software [48] and plotted with the OriginPro software [49].

### 2.4. Measurement Setup (Spinning Cell)

**Figure 4:**
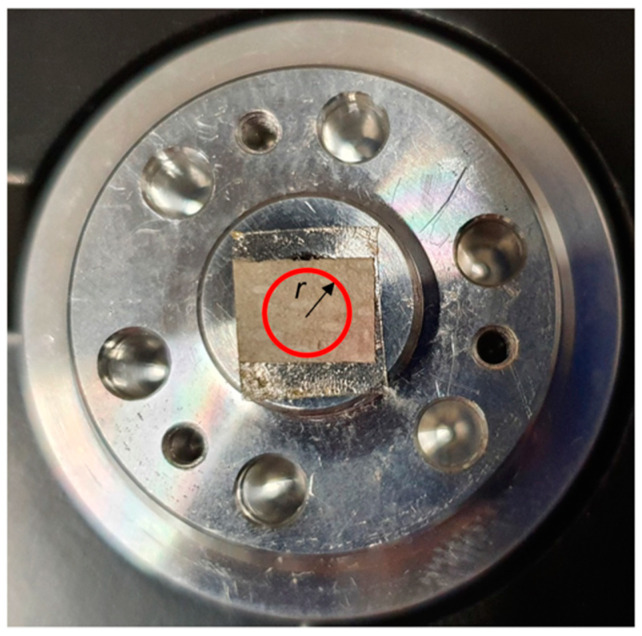
The spinning-cell setup. The substrate is mounted at the center of the rotating device and the laser is kept fixed at distance *r* from the rotation axis. During the SERS measurement, the laser track on the substrate follows the annular surface highlighted in red.

One of the main drawbacks of chemically synthesized SERS substrates is the lack of spatial uniformity, which implies high sensing variations across different points of the same substrate. The problem could be even more severe whit our paper-based SERS sensors: the paper support itself has an inhomogeneous texture (Figure 2), and the three-dimensional structure of the fibers can foster the accumulation of NPs in localized regions of the substrate. Indeed, both the substrates produced by centrifugation and those obtained by natural sedimentation display a highly non-uniform distribution of Ag NPs (Figure 3). In previous works [7,8], a practical and easy procedure was introduced to obtain spatially averaged SERS spectra on non-uniform substrates in short times. A rotating Hard Disk Drive (HDD) was used to spin the substrates, while the objective and the laser beam were kept fixed during the experiment. In this work, we adopt the single-centered drop spinning cell approach, consisting of placing one substrate at the center of the rotating device (Figure 4). The rotation angular frequency of the device is fixed and equal to 7200 RPM (754 rad/s), and the laser beam is off centered with respect to the rotation axis. As a result, the laser–sample interaction during the mechanical rotation of the substrate occurs along a circular path, and the Raman signal is collected from different points, leading to a net spectrum that is the spatial average of all the points probed during the measurement along the circular path. In addition to the spatial average of the SERS signal, another remarkable benefit of the spinning-cell setup is the reduced residence time of the laser at any point, which straightforwardly allows increasing the laser intensity with no risk of photo-induced damage. Simple kinematic analysis [8] shows that for a given angular speed, the more the laser spot is eccentric with respect to the axis of rotation, the more efficient the spinning cell approach will be compared to the static mode, both in terms of reduced exposure time on each spot along the circular path and in terms of an increased number of sample spots that are averaged out.

### 2.5. Perampanel Acidic Solutions

It has been reported that SERS of PER is fostered by protonation in acidic environments [6]. Therefore, we carried out the preparation of the PER aqueous solutions at variable concentrations (1 × 10^−4^ M, 1 × 10^−5^ M, and 1 × 10^−6^ M) in a controlled acidic solution, starting from a stock solution of 1 × 10^−3^ M PER in methanol. We prepared 3 mL of an acidic solution at pH 0 by mixing 25 μL of HCl 37% (Sigma-Aldrich) with 150 μL of H_2_SO_4_ 96% (Carlo Erba; Via R. Merendi 22, 20007 Cornaredo, Milan, Italy) and with 2.825 mL of distilled water. We diluted 50 μL of the solution at pH 0 with 4.95 mL of distilled water to obtain 5 mL of an acidic aqueous solution at pH 2, with a molar ratio HCl:H_2_SO_4_ of 1:9. The final step to prepare 1 mL of PER solution at the desired concentration was carried out by mixing selected volumes of PER 1 × 10^−3^ M solutions in suitable volumes of the acidic aqueous solution previously prepared (e.g., 1 mL [PER 1 × 10^−4^ M] = 0.1 mL [PER 1 × 10^−3^ M methanol soln.] + 0.9 mL [pH 2 water soln.]).

## 3. Results and Discussion

### 3.1. PER Detection

**Figure 5:**
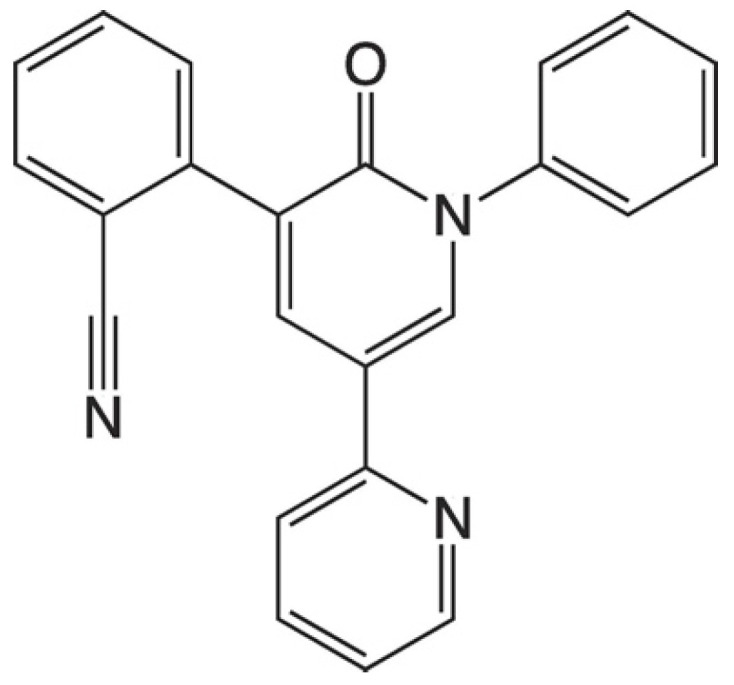
Molecular structure of PER.

The molecular structure of PER is reported in Figure 5. In reference [6], both solid PER and PER solutions were characterized by Raman spectroscopy to identify the main molecular markers and to investigate the best conditions for SERS sensing on Ag substrates produced by PLAL. The protonation of PER by acids (pH < 3) was proved by UV-Vis spectroscopy, and it was identified as a successful way to increase the interaction with the metallic substrate and to foster the SERS activity of PER [6]. Interestingly, in similar acidic environments, PER can be sensed by SERS also through Au substrates produced by PLD, as discussed in [10]. The following SERS markers of PER have been reported: 666 cm^−1^ (collective in-plane C-H bending), 877 cm^−1^ (collective out-of-plane C-H bending of the three outer rings), 1000 cm^−1^ (trigonal ring deformation of the three outer rings), 1599 cm^−1^ (ring stretching), and 2231 cm^−1^ (C≡N stretching) [6,10]. Further details about the assignment of PER bands can be found in references [6,17]. In addition to experimental error, slight variations in the peak position, typically in the range of a few wavenumbers, are ascribed to the different metal or to the different local chemical environment. The use of a 1:9 mixture of HCl:H_2_SO_4_ for setting the pH 2 environment suitable for SERS of PER [6] is required to reach the right acidification condition while reducing the total amount of chloride ions. Indeed, chlorides are essential to effectively reduce the background contribution due to citrate anions, but their excess may hinder the electrostatic interaction between protonated PER and the metal nanostructures.

**Figure 6:**
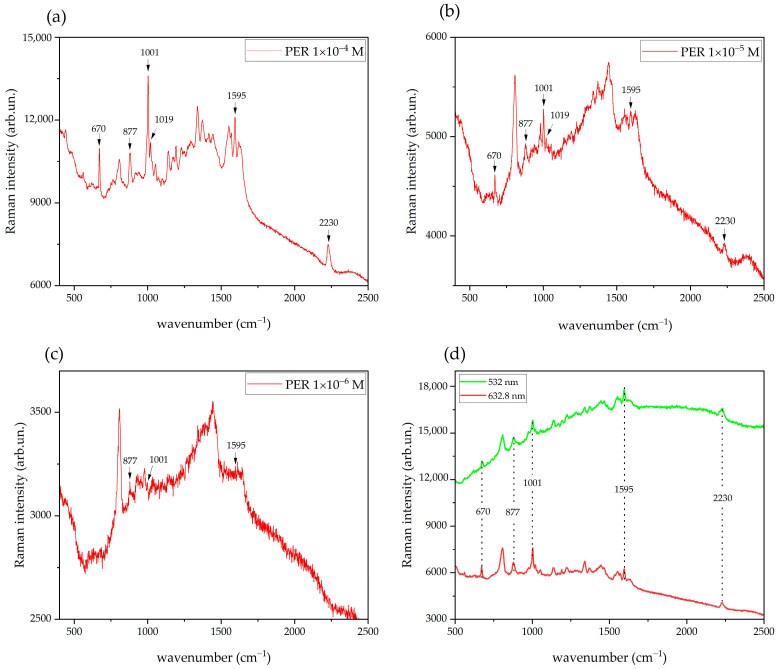
(**a**–**c**) SERS spectra of acidified PER in the range 1 × 10^−4^ M–1 × 10^−6^ M recorded with 632.8 nm excitation, 20× objective, 5 mW incident power, 10 s exposure time (5 averages), and spinning cell setup. (**d**) SERS spectra of acidified PER 1 × 10^−4^ M, recorded with 532 nm (3 mW) and 632.8 nm (5 mW) excitation wavelengths, 20× objective, 10 s exposure time (5 averages), and spinning cell setup. All the spectra are reported with no background subtraction and no normalization.

We tested the SERS substrates produced by centrifugation using 10 μL droplets of PER solutions (pH 2) at concentrations 1 × 10^−4^ M, 1 × 10^−5^ M, and 1 × 10^−6^ M (Figure 6). We acquired the spectra in spinning mode on the dried substrates. The comparison of the SERS signals obtained with the two excitations (at PER 1 × 10^−4^ M, Figure 6d) shows a lower fluorescence background for the 632.8 nm radiation. Therefore, to avoid the fluorescence background that originates from the support paper material, we selected the 632.8 nm excitation for all the SERS experiments discussed later.

All the expected SERS markers of PER (670, 877, 1001, 1019, 1595, 2230 cm^−1^) are well-resolved, intense, and clearly distinguishable from the spectral features of the background, which can be easily inferred from the SERS spectrum of PER measured at the lowest concentration (1 × 10^−6^ M, Figure 6c). In the spectrum of the solution at highest concentration (1 × 10^−4^ M) we can spot additional weak peaks at 1225 and 1447 cm^−1^ (in-plane C-H bending coupled with ring deformation [6]). The SERS spectrum of the 1 × 10^−6^ M solution (Figure 6c) shows some weak peaks that can be identified around 877, 1001, and 1595 cm^−1^, but the signal-to-noise ratio is too low to reliably quantify PER.

### 3.2. Overcoming the Spatial Non-Uniformity of the Paper-Based SERS Substrates by the Spinning Cell Apparatus

**Figure 7:**
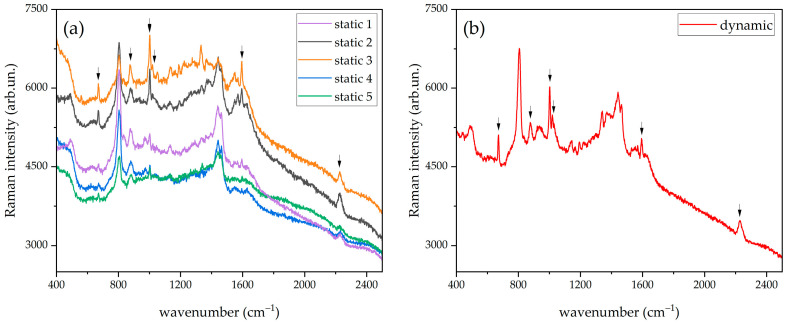
(**a**) SERS spectra of PER 1 × 10^−4^ M recorded in static mode in different spots or (**b**) with the spinning-cell setup (dynamic mode). 632.8 nm excitation laser, 5 mW incident power, 10 s exposure time (5 averages), 20× objective. All the spectra are reported with no background subtraction and no normalization. The main PER peaks are highlighted by arrows.

We first investigated the spatial uniformity of the SERS signal scattered by a representative SERS substrate produced by centrifugation by collecting the SERS spectrum in five randomly selected spots spanning over the whole substrate. The SERS substrate was prepared by depositing on it a droplet (8 μL) of acidified 1 × 10^−4^ M PER solution. The five SERS spectra were recorded after complete drying and are reported in Figure 7a using a common scale for the intensity of all the curves. The main PER peaks are visible in correspondence of each probed spot of the SERS substrate, but sizeable differences in peak intensity are observed from point to point. In addition, the contribution from the background, the signal-to-noise ratio, and the peak intensity ratios change from point to point. This behavior of the SERS substrate is of course detrimental in the development of sensing applications. However, as proved by the plot presented in Figure 7b, by collecting the SERS spectrum of the same substrate in spinning mode, we can significantly improve the signal-to-noise ratio, ease the identification of the marker peaks of PER, and efficiently average out the effects that local chemical variations and thermal fluctuations may have on the collected spectra. The spatial averaging, which is intrinsic with the spinning mode, also allows overcoming straightforwardly the issue of the substrate non-uniformity that was evidenced both by optical (Figure 2) and electron microscopy (Figure 3).

### 3.3. Quantitative SERS Measurements by the Spinning Cell

**Figure 8:**
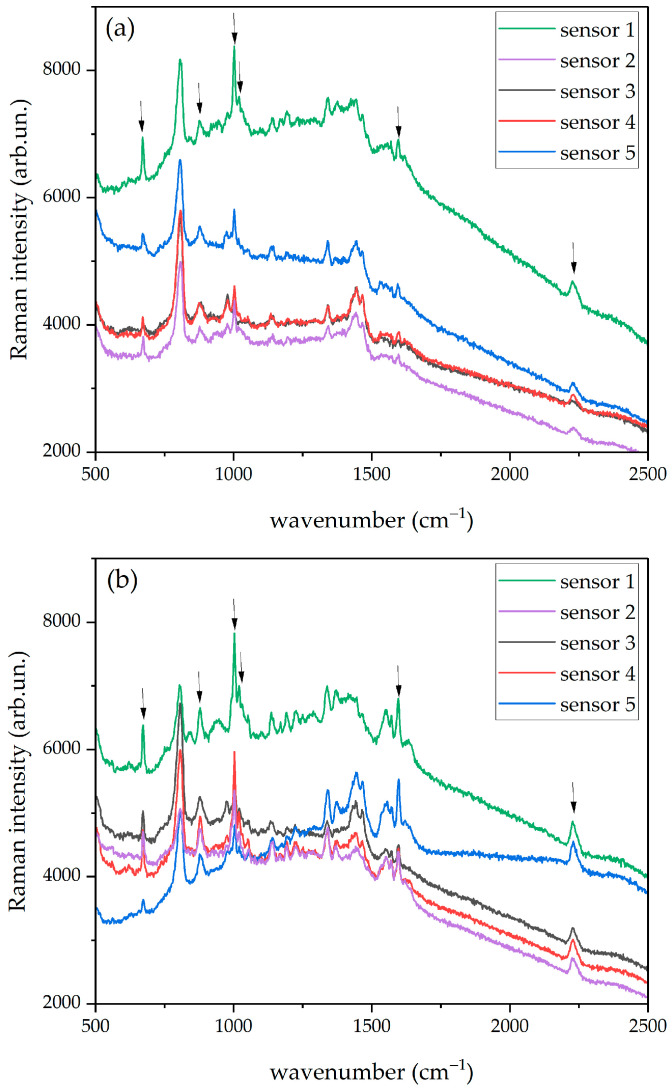
Representative set of SERS spectra (PER 5 × 10^−5^ M) recorded in dynamic mode on five different sensors produced either by (**a**) natural sedimentation or (**b**) centrifugation. 632.8 nm excitation laser, 5 mW incident power, 10 s exposure time (5 averages), 20× objective. The spectra are plotted with intensities in common scale. All the spectra are reported with no background subtraction and no normalization. The main PER peaks are highlighted by arrows.

**Figure 9:**
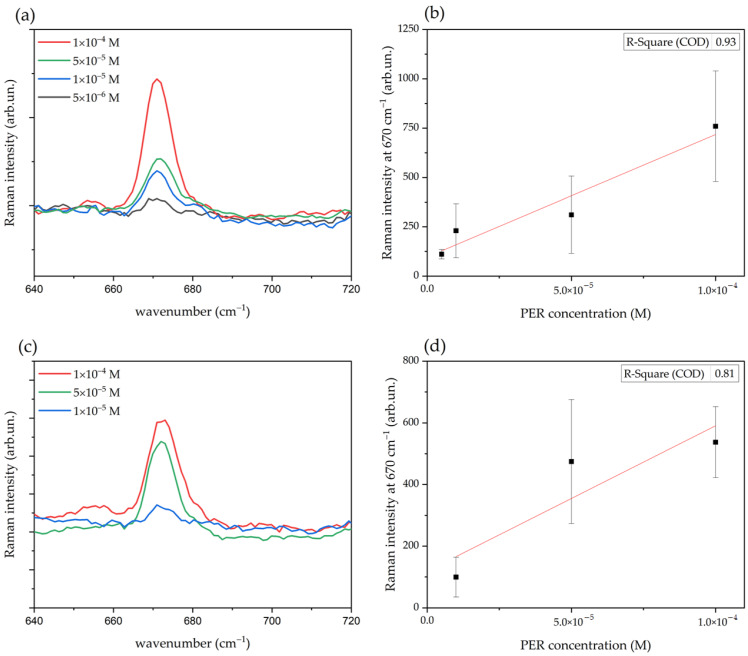
Baseline-corrected average SERS spectra of the 670 cm^−1^ marker of PER measured at different concentrations with sensors produced by natural sedimentation (**a**), or by centrifugation (**c**). For each specified concentration, the average spectrum is obtained from five spectra using the corresponding Omnic™ tool [48]. The panels (**b**,**d**) show the calibration curves corresponding to the SERS data (**a**,**c**), respectively; the vertical error bars represent the standard deviations.

Driven by the encouraging results that could be obtained by the spinning cell, we collected the SERS spectra in such dynamic mode on substrates produced by both natural sedimentation and centrifugation (for the quantitative measurements reported in Figure 8, Figure 9 and Figure SERS substrates by centrifugation were produced following the centrifuge-assisted procedure reported in Section 2.2 but using 1 mL of concentrated Ag colloid). We used for each measurement 8 µL of PER solution, and for each PER concentration, we repeated the measurement on five different sensors. We prepared the aqueous solutions at four different PER concentrations (1 × 10^−4^ M, 5 × 10^−5^ M, 1 × 10^−5^ M, 5 × 10^−6^ M) in acidic conditions (pH 2) starting from proper volumes of a stock solution of PER 1 × 10^−3^ M in methanol (see the Experimental section). We prepared the acidified PER solutions just before the measurements as a precaution to avoid possible degradation issues of the PER molecule and ensure reliable SERS detection.

We report in Figure 8a,b two selected sets of SERS spectra obtained from PER 5 × 10^−5^ M solutions that were recorded, respectively, on paper-based SERS sensors produced by natural sedimentation and by centrifugation (see Section 2.2). Six PER markers (670, 877, 1001, 1019, 1595, and 2230 cm^−1^) are clearly visible in both cases. Similar results are obtained for PER 1 × 10^−4^ M and 1 × 10^−5^ M. The intense peak around 807 cm^−1^, which is not affected by PER concentration, is assigned to the background, together with the structured feature between 1300 and 1500 cm^−1^.

At the PER concentration of 5 × 10^−6^ M (which was tested only on sensors produced by natural sedimentation), the PER peak around 1019 cm^−1^ is no more visible, while the PER peaks at 1595 cm^−1^ and 2230 cm^−1^ are very weak and not always detectable. Although in such conditions, some features assigned to PER are still unambiguously detected in the SERS spectra and we can safely take the concentration value of 5 × 10^−6^ M as the lowest limit of detection for reliable PER detection with this type of substrate.

We assessed the calibration curves of both types of paper-based SERS sensors over the PER concentration range between 1 × 10^−4^ M and 5 × 10^−6^ M (sensors produced by natural sedimentation) and between 1 × 10^−4^ M and 1 × 10^−5^ M (sensors produced by centrifugation). The intensities reported in Figure 9 have been obtained as peak height with respect to the baseline and were determined by using the peak height tool of the Omnic™ software [48]. For each concentration, the SERS intensity data have been collected on five spectra, and the average value was used to determine the linear regression plotted in Figure 9. We have considered the four peaks that are clearly visible and easily resolved at any concentration, namely 670 cm^−1^, 877 cm^−1^, 1001 cm^−1^, and 2230 cm^−1^. We report in Figure 9 the calibration curves for the representative PER peak at 670 cm^−1^, for both types of substrates (produced by natural sedimentation and centrifugation), along with the corresponding concentration-dependent baseline-corrected average SERS spectra.

As expected, the average intensity displays an increasing trend with concentration, which can be fitted by a linear regression with acceptable values of the coefficients of determination R^2^. Nevertheless, for the same PER marker and concentration, we observe quite a large variability of intensity across different SERS sensors, as can be inferred by Figure 8 and as clearly underlined by the error bars of Figure 9. Therefore, in view of analytical applications, we believe that the inter-sample repeatability and reproducibility should be improved by properly engineering the fabrication process, so as to minimize uncontrolled changes in the physical-chemical conditions of the deposition and aggregation phenomena of the NPs on the paper fibers.

We also tested the stability of the SERS signal of the sensors over time. To this aim, we collected the SERS spectra of PER 1 × 10^−4^ M (on substrates produced by both natural sedimentation and by centrifugation) 36 h after the deposition of the PER solution on the sensors, and we compared such spectra against the SERS spectra recorded 15 min after the deposition (with the same experimental parameters). We observed no significant variations, and the SERS signal was still good after 36 h, especially for the substrates produced by centrifugation.

## 4. Conclusions

In conclusion, by depositing Lee-Meisel Ag colloids on filter paper, we prepared SERS substrates by a fast, simple, and inexpensive approach. In all the substrates, long and thin nanorods, along with large assemblies of closely packed NPs, are responsible for the sensing properties of sensors produced by both natural sedimentation and by centrifugation. Such nanostructures are spatially not uniformly distributed but tend to accumulate in cavities and voids among the paper fibers. By spinning at 7200 RPM such paper-based SERS sensors with a custom device obtained from a hard disk drive, the anti-epileptic drug PER could be clearly detected in the concentration range between 1 × 10^−4^ M and 5 × 10^−6^ M, and high-quality spectra were obtained. We assessed the calibration curves of the average baseline-corrected peak heights, linearly correlated to the molar concentration of PER in solution. Further optimization of the production process should aim at reducing the hindrance caused by the background signal that overlaps with the SERS signals of the analyte and therefore limits the minimum detectable concentration. To this aim, one should test other kinds of paper supports that could be less prone to strong background contributions that may arise from the paper itself or industrial additives. Furthermore, also the change of the reducing agent, together with the in situ reduction of noble metals at the paper surface [28,33,34] could significantly improve the reproducibility of the production (avoiding the colloid deposition step) and the spatial uniformity of the coverage of the NPs on the support. Finally, the use of excitation wavelengths closer to the plasmon peak of the colloid (e.g., 405 nm) could significantly improve the signal enhancement in the case of Ag-based sensors. By properly addressing such issues, one could investigate SERS sensing of PER within the therapeutic range with highly reproducible paper-based SERS substrates.

## Data Availability

The data presented in this study are available in this article.
